# Defining the dimensions of circulating tumor cells in a large series of breast, prostate, colon, and bladder cancer patients

**DOI:** 10.1002/1878-0261.12802

**Published:** 2020-10-04

**Authors:** Pauline A. J. Mendelaar, Jaco Kraan, Mai Van, Leonie L. Zeune, Leon W. M. M. Terstappen, Esther Oomen‐de Hoop, John W. M. Martens, Stefan Sleijfer

**Affiliations:** ^1^ Department of Medical Oncology Erasmus MC University Medical Center, Cancer Institute Rotterdam The Netherlands; ^2^ Department of Medical Cell BioPhysics University of Twente Enschede The Netherlands

**Keywords:** cell morphology, cell size, circulating tumor cells, metastatic cancer, single cell isolation methods

## Abstract

Circulating tumor cells (CTCs) in the blood of cancer patients are of high clinical relevance. Since detection and isolation of CTCs often rely on cell dimensions, knowledge of their size is key. We analyzed the median CTC size in a large cohort of breast (BC), prostate (PC), colorectal (CRC), and bladder (BLC) cancer patients. Images of patient‐derived CTCs acquired on cartridges of the FDA‐cleared CellSearch^®^ method were retrospectively collected and automatically re‐analyzed using the accept software package. The median CTC diameter (μm) was computed per tumor type. The size differences between the different tumor types and references (tumor cell lines and leukocytes) were nonparametrically tested. A total of 1962 CellSearch^®^ cartridges containing 71 612 CTCs were included. In BC, the median computed diameter (CD) of patient‐derived CTCs was 12.4 μm vs 18.4 μm for cultured cell line cells. For PC, CDs were 10.3 μm for CTCs vs 20.7 μm for cultured cell line cells. CDs for CTCs of CRC and BLC were 7.5 μm and 8.6 μm, respectively. Finally, leukocytes were 9.4 μm. CTC size differed statistically significantly between the four tumor types and between CTCs and the reference data. CTC size differences between tumor types are striking and CTCs are smaller than cell line tumor cells, whose size is often used as reference when developing CTC analysis methods. Based on our data, we suggest that the size of CTCs matters and should be kept in mind when designing and optimizing size‐based isolation methods.

## Graphical abstract

Molecular characterization of circulating tumor cells is of high clinical relevance. Since circulating tumor cell (CTC) detection and isolation often rely on cell dimensions, we determined the size of 71 612 CellSearch‐detected CTCs using accept software. Strikingly, CTC size differs between tumor types and significantly deviates from the size of cultured tumor cells, which is currently used in the development of CTC isolation methods.

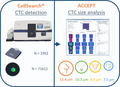

**Abbreviations**
ACCEPT: Automated CTC Classification, Enumeration, and PhenoTyping software
BC: breast cancer
BLC: bladder cancer
CD: computed diameter
CEL: cultured tumor cell (cell line)
CK: cytokeratin
CRC: colorectal cancer
CTC‐L: circulating tumor cells derived from cerebrospinal fluid (liquor)
CTCs: circulating tumor cells
DAPI: 4′6‐diamidino‐2‐phenylindole
EMT: epithelial–mesenchymal transition
EpCAM: epithelial cell adhesion molecule
IQR: interquartile range
KW test: Kruskal–Wallis test
MWU test: Mann–Whitney U test
NCR: nucleus/cytoplasm ratio
P2A: perimeter to area
PC: prostate cancer
TIF: tagged Image Format files
TXT: text file
μm: micrometer
µm^2^: square micrometers

## 1 Introduction

Circulating tumor cells (CTCs) are disseminated from solid malignancies and present in the peripheral circulation of patients of multiple tumor types [1]. They are thought to originate from all tumor lesions present in the body. Therefore, CTCs could reflect a sample of the molecular landscape of the disease in real time when successfully captured and characterized. Because the median concentration in patients with metastatic disease is estimated as 1 CTC per billion white blood cells, capturing them is technically challenging. This emphasizes the need for sensitive and specific CTC detection and isolation methods.

The CellSearch^®^ system is the only FDA‐cleared CTC enumeration platform [2]. This method uses antibodies recognizing the epithelial cell adhesion molecule (EpCAM) coupled to ferrofluids to enrich peripheral blood for CTCs. The immunomagnetically enriched cells are stained with the nucleic acid dye 4′6‐diamidino‐2‐phenylindole (DAPI) and labeled with fluorescent antibodies against cytokeratins 8, 18+, and/or 19+ (CK) and CD45. The processed blood fraction is transferred into a cartridge of which images are taken. An expert user of the CellSearch^®^ method assesses all presented events and identifies CTCs manually. CTCs are defined as CK+/DAPI+/CD45− events, whereas leukocytes are CK−/DAPI+/CD45+ events. CellSearch‐based CTC enumeration has prognostic value in multiple tumor types [3, 4, 5]. However, since the method relies on EpCAM positivity, it will only detect CTCs which express EpCAM. Epithelial–mesenchymal transition (EMT) is thought to play a key role in dissemination of cancer [6, 7]. To a certain degree, epithelial markers are down regulated in CTCs which have undergone EMT. This can lead to a detection failure using the CellSearch^®^ method if EpCAM expression is too low or absent. Hence, EpCAM‐based detection methods may introduce a selection bias in CTCs which can be studied.

Therefore, antibody‐independent CTC detection and isolation methods are being developed and many of them are based on physical characteristics [8, 9, 10, 11, 12, 13]. Size‐based methods frequently use cultured cell line cells to characterize the performance of their system. Although evidence is lacking, CTCs are considered to be larger and less deformable than blood cells. Additionally, it is suggested that morphological differences of CTCs exist between tumor types [14, 15]. This implies the need for improved knowledge on CTC size from different tumor types. Therefore, we determined the median CTC size, computed the approximate median diameter (CD) of the cytoplasm and nucleus per cell, and determined the nucleus/cytoplasm ratio (NCR) of CTCs from breast (BC), prostate (PC), colorectal (CRC), and bladder (BLC) cancer patients. For this purpose, a large series of images of CellSearch^®^ cartridges were re‐analyzed through the ‘Automated CTC Classification, Enumeration and PhenoTyping software package’ (accept) (https://github.com/LeonieZ/ACCEPT) [16]. In addition, we compared the CTC results to data derived from lymphocytes and well‐known cancer cell lines.

## 2 Materials and methods

### 2.1 Data collection/CellSearch^®^

CellSearch^®^ CTC enumeration is part of ongoing clinical research of the medical oncology department at the Erasmus Medical Center, Rotterdam, the Netherlands. Here, we retrospectively studied the images of CellSearch^®^ cartridges of BC, PC, CRC, and BLC patients. All patients provided written informed consent and were included in clinical trials designed in accordance with the Helsinki Declaration and approved by the local ethics board of the Erasmus MC University Medical Center (Table S1). Subject numbers, cartridge numbers, and CellSearch^®^ appointed CTC counts were collected for all patients. To acquire images of cultured tumor cells, cartridges of experiments in which three BC cell lines (MCF‐7, SKBR3, and TD47D) and one PC cell line (LNCAP) were spiked into tubes of blood of healthy donors were studied. Leukocyte analysis was enabled through the selection CD45+/DAPI+/CK− events on one melanoma patient‐derived cartridge. CellSearch^®^ generated data consisting of a maximum of 175 Tagged Image Format files (.TIF) per sample, an Extensible Markup Language (.xml) containing the coordinates of the manually marked CTCs, were acquired per cartridge. The images were annotated by tumor type, patient, and material of origin (blood vs liquor). Data collection took place between January 2017 and January 2019.

### 2.2 Data processing/accept and spss statistics 25

accept is an open source program developed at the University of Twente, which enables the re‐analysis of images of CTCs acquired through the CellSearch^®^ method. [16]. Here, collected CellSearch^®^ images were re‐analyzed using the ‘marker characterization mode’ of accept. This software feature enables the analysis of only those events which were originally defined as CTCs by an expert user of the CellSearch^®^ platform. accept used the coordinates of marked events present in the CellSearch^®^ generated .xml file to trace back CTCs and selected spiked tumor cells and leukocytes. The program automatically detects the immunofluorescent signals present in the DAPI, CK, and CD45 channel and marks the borders of these events. Per individual event, accept reports multiple parameters of roundness, signal intensity, and size for the DAPI, CK, and CD45 channel per cartridge in an extended excel file (Microsoft Excel v.10, 365 Office; Microsoft, Redmond, WA, USA). Subject numbers, tumor type, and type of material (blood vs liquor) were added manually per included study. Subsequently, the excel files containing accept data of all cartridges were combined into one ibm spss statistics 25 file [International Business Machines Corporation (IBM), Armonk, NY, USA].

### 2.3 Data analysis

Descriptive statistics on the origin of the cartridges, and CTC counts were collected from different data files. CellSearch^®^ selected CTC counts were derived from prior clinical study files (excel). To determine the ability of accept to represent the CellSearch^®^ marked events, accept enumeration results were compared to the known CellSearch^®^ CTC counts. All following analyses were performed using the spss file containing multiple variables per event.

### 2.4 Selection of cohort for CK and DAPI size analysis

To examine the extent to which the reported accept events met the CellSearch^®^ CTC criteria, CK and DAPI positivity were assessed per event. All criteria mentioned were applied to patient‐derived CTCs as well as cell line cells. To adequately determine the size of CTCs per tumor type, it was important to analyze only those events containing confirmed single CTCs. Therefore, events which entered the size analysis had to meet two selection criteria to correct for any inaccuracies introduced by the accept processing. First, accept reported events had to have a positive CK signal. Importantly, the CellSearch^®^ marked CTC coordinates in the .xml file could contain images of multiple CK positive CTCs. Therefore secondly, a prior described method based on the ‘perimeter to area’ (P2A) ratio of the CK channel was used to discriminate between single CTCs, doublets, and small and large clusters to enable the selection of single CTCs for further analyses [17]. Only single, CK positive events were included in the size analysis. To enable the analysis of leukocytes, the CK selection criteria were applied to the CD45 signal.

Single CK‐positive events had to meet three additional criteria to analyze the cytoplasm/nucleus ratio (NCR) from each CTC. First, as accept did not detect a DAPI signal in all single CK positive CTCs, the DAPI signal had to be positive. This was defined as the DAPI size being > 0 μm^2^. Secondly, the P2A measurement of the DAPI signal was assessed to select only those CTCs with a single nuclear signal. The third criterion to enter the analysis was that the nucleus had to be intact. This was defined by the fact that the DAPI signal (µm^2^) had to be smaller than the CK signal (µm^2^).

### 2.5 CK and DAPI size determination and cytoplasm/nucleus assessment

The size of the CK and DAPI signals in square micrometers (µm^2^) as reported by accept was used to compute an approximate diameter of the cytoplasm and nucleus per CTC. The median size of the predefined CK‐positive single CTCs was calculated per tumor type. To assess whether a difference in CK and or DAPI size existed between the four tumor types, the Kruskal–Wallis test (KW test) was used. Subsequently, when a significant size difference existed, the Mann–Whitney *U* test (MWU test) was applied pairwise to assess the size differences between the tumor types. Within the BC CTCs, the MWU test was also used to assess the size difference between blood‐ and liquor‐derived CTCs. Additionally, for BC and PC, the size difference between patient‐derived CTCs and tumor cell line cells was assessed using the MWU test. Finally, this test was used to compare the size of leukocytes to the size of blood‐derived CTCs of all tumor types. The computed DAPI and CK diameters were used to determine the NCR in CK positive single CTCs, which had an intact, single positive DAPI signal. The nuclear size and NCR differences between tumor types were assessed through the same nonparametrical testing as mentioned above.

## 3 Results

### 3.1 CellSearch^®^ cartridges – Input data description

A total of 1962 CellSearch^®^ cartridges from 1419 individuals were included. The images of 970 BC (655 individuals), 275 PC (185 individuals), 416 CRC (280 individuals), and 301 BLC cartridges (299 individuals) were re‐analyzed (Table 1, Figs S1 and S2). Within the BC cohort, 43 of the 970 cartridges concerned liquor‐derived images. According to the original CellSearch^®^ data, 66.3%, 84.0%, 43.9%, and 25.2% of the BC, PC, CRC, and BLC cartridges contained CTCs, respectively. From the liquor‐derived BC cartridges, 34.9% were CellSearch^®^ CTC positive. Assessment of the original CellSearch^®^ CTC counts showed that for BC, a total of 48 242 blood‐derived (CTC‐B) and 3285 CTCs derived from cerebrospinal fluid [CTC‐L(iquor)] CTCs were included. In PC, this was 20 442, whereas in CRC and BLC the input CTC count was significantly lower; 626 and 260, respectively (Table 1). Median CTC counts per cartridge were 2 [Interquartile range (IQR) 0–11], 11 (IQR 2‐54), 0 (IQR 0‐1), and 0 (IQR 0‐1) for BC, PC, CRC, and BLC, respectively. The maximum CTC counts were 3254 for BC, 7333 for PC, 51 for CRC, and 32 for BLC samples.

**Table 1:** Description of CellSearch^®^ input data.

	Number of cartridges (CTC+)	Number of patients (CTC+)	Number of CTCs
Breast			
Total	970 (643)	655 (497)	51 527
Blood	927 (628)	655 (497)	48 242
Liquor	43 (15)	35 (11)^a^	3285
Prostate			
Blood	275 (231)	185 (168)	29 738
Colorectal			
Blood	416 (183)	280 (136)	626
Bladder			
Blood	301 (76)	299 (76)	260

^a^ Patients also represented in the ‘breast‐blood sample cohort’, therefore not unique.

All reference cell line cells were spiked in healthy donor blood and processed by the CellSearch^®^ method. Regarding the BC cell line data, three cartridges containing SKBR3 cells, two cartridges with MCF‐7 and one TD47D cells resulted in 530, 1616, and 1249 evaluable events, respectively. One PC cartridge resulted in 1054 LNCAP cells. Selection of leukocytes in one cartridge of a melanoma patient resulted in 130 evaluable events (Table S2).

### 3.2 accept output—data description and optimization cohort for CK and DAPI size analysis

accept detected a total of 72 840 events distributed over the four tumor types. In BC, the number of detected events in blood‐derived cartridges was 45 695 and 5327 in liquor‐derived cartridges. As summarized in Fig. S3 and Table S3, 99.5% of the events detected in blood and 99.9% of the events detected in liquor were CK positive, while selecting for both CK and DAPI positivity resulted in 86.2% and 99.1% of the events in blood and liquor, respectively. In PC, 20 624 events were detected of which 97.5% was CK positive and 89.3% was CK and DAPI positive. As seen in Fig. S4, the analysis of CRC cartridges resulted in the detection of 934 events of which 55.6% was CK positive and 47.32% was CK and DAPI positive. Finally, 260 events were detected in the BLC samples, of which 76.2% was CK positive and 72.7% was CK and DAPI positive.

**Fig. 1:**
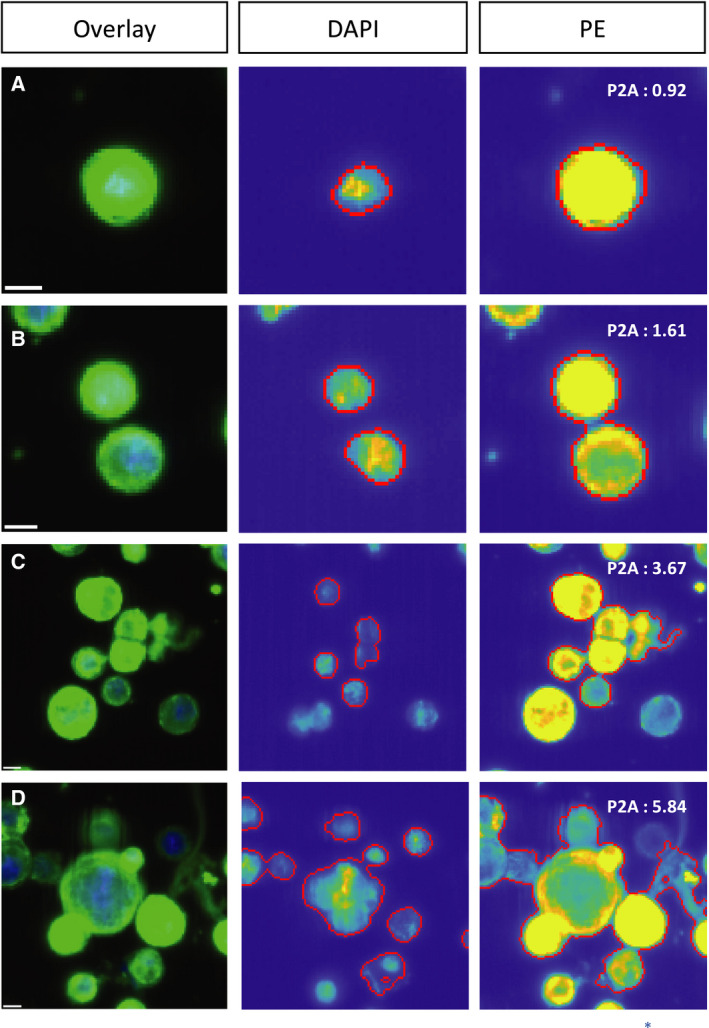
Event types ACCEPT defined by PE‐P2A. (A) Single CTC (P2A < 1.5), (B) doublet (P2A ≥ 1.5 < 2.5), (C) small CTC cluster (P2A ≥ 2.5 < 4), (D) large CTC cluster (P2A ≥ 4). Scale bar: 6.4 μm.

Singularity determination of the CK‐positive events resulted in 44 232 single blood‐derived BC CTCs (97.3%), 1172 doublets, 68 small clusters, and 1 large cluster. In the re‐analyzed cartridges containing liquor‐derived BC samples, 4702 CTCs (88.4%) were single, while 513 doublets, 104 small clusters, and 3 large clusters occurred. In PC, 19 117 single CTCs (95.1%) as well as 916 doublets, 63 small, and 4 large clusters were found. Within CRC, this resulted in 503 single CTCs (96.9%), 12 doublets and, 3 small and 1 large cluster. Finally, in BLC, 182 of the CK positive events (91.9%) were single CTCs, while 15 doublets and 1 small cluster occurred (Table 2, Fig. 1).

**Table 2:** CTC counts per tumor type and type of event. Singularity determination of CK+ accept events through the use of the CK P2A results.

	Material	Single CTC	Doublet	Small cluster	Large cluster	Total
Breast	Blood	44 232	1172	68	1	45 472
	Liquor	4702	513	104	3	5322
Prostate	Blood	19 117	916	63	4	20 100
Colorectal	Blood	503	12	3	1	519
Bladder	Blood	182	15	1	0	198
Total		68 736	2628	239	9	71 612

Assessment of the 48 934 positive single CTCs in BC samples resulted in a total of 38 918 DAPI evaluable events (DAPI+ and DAPI signal < CK signal). Of these events, 38 211 showed a single DAPI signal, 682 showed a double signal and 25 showed a clustered signal. Within the 19 117 single CTCs in the PC samples, 13 356 were DAPI evaluable of which 13 127 were single, 224 double, and 5 were clustered DAPI signals, respectively. Out of the 503 single CRC CTCs, a total of 255 events were DAPI evaluable of which 252 had a single DAPI signal and 3 events had a double signal. Finally, from the 182 single BLC CTCs, the DAPI signal was evaluable in 99 events, resulting in 92 times a single signal, 4 times double, and 2 times a clustered signal (Table S4).

### 3.3 accept output—Analysis of size and nucleus/cytoplasm ratio

**Fig. 2:**
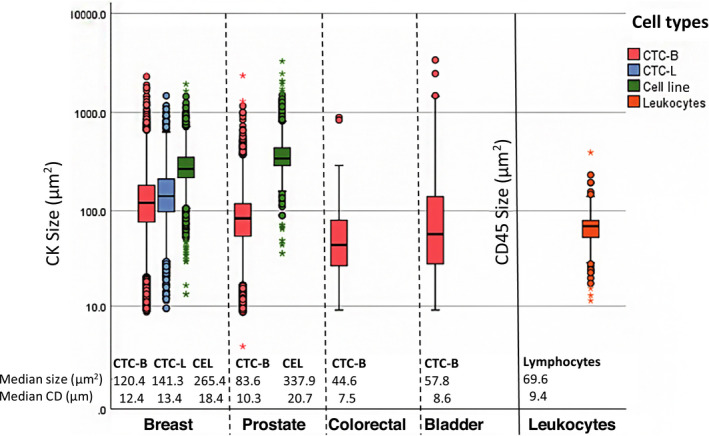
Cell size (μm^2^) per cell type. Included accept events: CK+, single events. Breast cancer: *n* = 44 232 CTC‐B (blood‐derived CTCs) and *n* = 4702 CTC‐L (liquor‐derived CTCs). Prostate cancer: *n* = 19 117 CTCs. Colorectal cancer: *n* = 503 CTCs. Bladder cancer: *n* = 182 CTCs. CEL: cell line cell; included events: CK+ single cultured breast cancer cells [MCF‐7 (*n* = 1616); SKBR3 (*n* = 530); TD47D (*n* = 1249)] and prostate cancer cells [LNCAP (*n* = 1054)], and CD45+ single lymphocytes (*n* = 130).

**Fig. 3:**
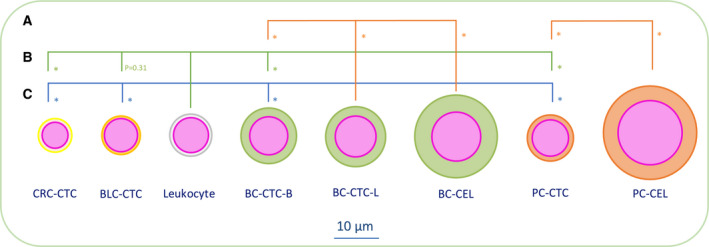
Statistical analysis of cell size differences. (A) MWU test CK CD difference within BC (CTC‐B vs CTC‐L and CTC‐B vs CEL) and PC (CTC vs CEL); *P < 0.001, (B) MWU test of CD between CTCs and leukocytes; *P < 0.001, (C) MWU test of CK CD of blood‐derived CTCs between the four different tumor types; *P < 0.001.

Figure 2 shows a median size of blood‐derived BC CTCs of 120.4 μm^2^ (IQR 104.9) [computed median diameter (CD): 12.4 μm]. Liquor‐derived BC CTCs had a median size of 141.3 μm^2^ (IQR 113.1, CD 13.4). The median size of cultured BC cells was 265.4 μm^2^ (IQR 127.38, CD 18.4 μm). In increasing order of size, the median size of the three BC cell lines was 241.7 μm^2^ (MCF‐7, IQR 106.5, CD 17.54 μm), (T47D, IQR 95.25, CD 18.35 μm), and (SKBR3, IQR 169.58, CD 22.01 μm) (See Figs S5 and S6 for specific cell line details). PC CTCs had a median size of 83.6 μm^2^ (IQR 63.5, CD 10.3 μm), while the median size of cultured PC cells was 337.9 (LNCAP IQR 142.5, CD 20.7 μm). The CTCs in CRC samples had a median size of 44.6 μm^2^ (IQR 53.3, CD 7.5 μm), and the CTCs in the BLC samples had a median size of 57.8 μm^2^ (IQR 111.5, CD 8.6 μm). Finally, analysis of the lymphocyte data resulted in a median size of 69.6 μm^2^ (IQR 25.6, CD 9.4 μm). When nonparametrical testing was applied (Fig. 3), the independent samples median test (*P* < 0.001) and the KW test (*P* < 0.001) showed an existing size difference within the patient‐derived CTCs of the different tumor types. When testing the size differences between two tumor types at a time, the MWU test resulted in *P* < 0.001 between every tumor type. MWU testing also showed that the median size difference of 20.9 μm between blood‐derived and liquor‐derived BC CTCs was significant (*P* < 0.001). Additionally, the median size differences between cell line cells and patient‐derived CTCs of 145 μm in BC and 254.3 μm in PC were both significant (*P* < 0.001). Finally, the MWU test was applied to assess statistical significance between the median size differences between leukocytes and BC (−50.8 μm, *P* < 0.001), PC (−14.0 μm, *P* < 0.001), CRC (+25 μm, *P* < 0.001), and BLC (−11.8 μm, *P* = 0.31).

**Fig. 4:**
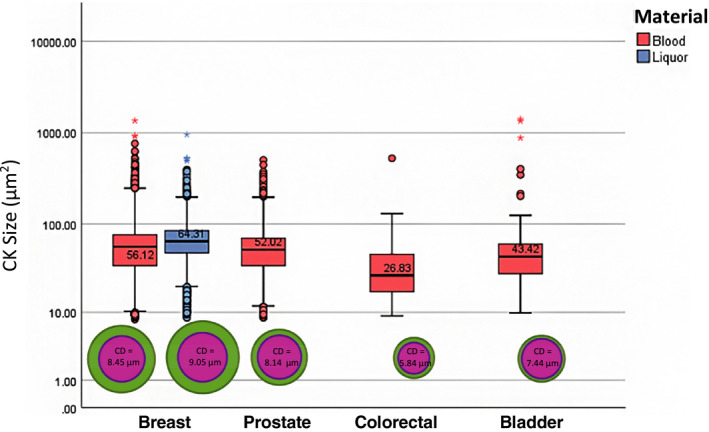
Nucleus computed diameter (μm) and size (μm^2^) per tumor type. Included: CK+, single CTC, single DAPI, DAPI < CK accept events. Breast cancer: *n* = 33 696 CTC‐B (blood‐derived CTCs) and *n* = 4515 CTC‐L (liquor‐derived CTCs). Prostate cancer: *n* = 13 127 CTCs. Colorectal cancer: *n* = 252 CTCs. Bladder cancer: *n* = 93 CTCs.

Figure 4 shows a median size of 56.1 μm^2^ (IQR 41.8) for the nucleus of the blood‐derived BC CTCs resulting in a NCR of 0.47. The median size of BC cell line cells was 94.2 μm^2^ (IQR 50, NCR 0.37). Both nucleus size and NCR were statistically significant between patient‐derived CTCs and BC cell line cells (*P* < 0.001). In liquor‐derived BC CTCs, the median size of the nucleus was 64.3 μm^2^ (IQR 36.9, NCR = 0.46). For PC CTCs, the median size of the nucleus was 52.0 μm^2^ (IQR 35.2, NCR = 0.62), while PC cell line cells had a median nucleus size of 161.0 μm^2^ (IQR 2.85, NCR = 0.48). Again, both the nucleus size difference as well as the NCR were statistically different between CTCs and cultured cells (MWU *P* < 0.001). The nucleus size of CRC CTCs was 26.83 μm^2^ (IQR 29.0, NCR = 0.60), and in BLC 43.4 μm^2^ (IQR 33.6, NCR = 0.75). Nonparametrical testing between the different tumor types was applied as stated above; the independent samples median test resulted in *P* < 0.001 and the KW test in *P* < 0.001. When testing the nuclear size differences between two tumor types at a time, the MWU test resulted in *P* < 0.001 between every tumor type except for the PC vs BLC analysis (*P* = 0.073).

## 4 Discussion

In this study, we assessed the median size of CTCs in a large cohort of BC, PC, CRC, and BLC patients using accept hypothesizing that there might be a difference in CTCs across tumor types and between patient‐derived CTCs and cell line cells, implying the need for tumor‐specific CTC isolation methods. We indeed found different median CTC sizes between the four tumor types with BC CTCs being the largest and CRC CTCs the smallest, respectively. Furthermore, we found that liquor‐derived BC CTCs are larger than blood‐derived CTCs in the same population of patients, suggesting a morphological difference between cells derived of the two types of origin. Finally, the median size of the nucleus also differed between the different tumor types although the variance was smaller than within the distribution of the CK signal.

Two prevailing dogmas regarding CTC size, which are highly important with respect to the future development of size‐based isolation methods, are addressed by the analysis of our patient‐derived CTC data in comparison with our reference data. The first widely accepted hypothesis states that CTCs are generally larger than white blood cells. Literature describes a median size of lymphocytes of 7.1–10.5 μm, and 8.7–9.9 μm for granulocytes [18], which implies that the CTC size as computed in this study is much more similar to the known size of white blood cells than postulated. Our data supports this hypothesis as the leukocytes we analyzed did not statistically differ from BLC CTCs and were even significantly larger than CRC CTCs. Secondly, the development of size‐based isolation methods heavily relies on spike experiments using cell line tumor cells, based on the assumption that their median size is comparable to that of CTCs. Our data show that patient‐derived CTCs are generally smaller than the cultured cell line cells of the respective tumor type as described in the literature. In BC, the CD of patient‐derived CTCs was 12.4 μm, compared to the larger, cultured cells which were 15–17 μm (SKBR‐3), and 16.5 μm (MCF‐7). In prostate cancer, the median CD of 10.3 μm is also significantly smaller than the size of a cultured cells: 18–21 μm (PC3‐9). This is also the case in colorectal cancer, where our computed median diameter is 7.5 μm vs 11 μm in cultured cells (SW480) [19]. Our analysis of three BC cell lines (median size of 18.4 μm) and one PC cell line (20.7 μm) were comparable to these data. With the results of this study, we show that the median size of cell line cells is indeed significantly larger than patient‐derived CTCs in both BC and PC.

When the results of our analysis were compared to the expected input data retrieved from the CellSearch^®^ data files, the efficiency of accept to analyze previously CellSearch^®^ marked events was high. However, the increased number of accept detected events compared to the CellSearch^®^ marked cell count in CRC was striking. A possible explanation is that cartridges of the specific clinical trial in which this occurred contained the images of the buffy coat of 30 mL blood instead of the usual 7.5 mL whole blood. Within this technique, 30 mL of blood is pooled into 2 × 15 mL tubes, after which plasma separation and the creation of a buffy coat is achieved through a centrifuging step. After pipetting off the blood plasma, the two buffy coats are pooled into one CellSave tube. After these preparation steps, the CellSearch technique is applied in the same manner as on 7.5 mL blood. It is possible that the high background of leukocytes in these samples led to a more difficult distinction for accept to recognize CTCs as a single event.

The main limitations of this study concern the introduction of a selection bias in the analyzed data. First, its retrospective nature led to the inclusion of a cohort of patients with a wide range of disease characteristics. The cartridge data of all patients included in one of the historical clinical trials were re‐analyzed, irrespective of the disease stage, metastatic status, and the treatment line or choice. This could lead to an under‐ or overestimation of the CTC counts within the current cohort. Second, as we made use of images acquired through the CellSearch^®^ method, the analysis is limited to EpCAM positive CTCs. Although this type of CTCs is of prognostic value in all studied tumor types, it is increasingly suggested that cells which have undergone epithelial‐mesenchymal transition (EMT) have a higher metastatic potential and are therefore of interest regarding their characterization. These EMT cells could not be studied through the current method. The third limitation regards the marker characterization mode of accept. We chose to analyze only those events which were previously marked as CTCs and therefore had to meet the CellSearch^®^ criteria of CK positivity, CK signal ≥ 4 μm^2^, DAPI signal which overlaps the CK signal for ≥ 50%, and a morphological appearance of an intact cell. It is possible that the studied CTC cohort reflects a certain population of cells due to this selection.

## 5 Conclusion

Although the re‐analyzed data in this study were subject to a certain degree of selection bias, to the best of our knowledge it does reflect the largest cohort of morphological studied CellSearch^®^ depicted CTCs which are directly compared to reference data. In addition, one could suggest that the size difference as found in this cohort, irrespective of a possible selection bias, reflects an even more pronounced difference when corrected for this limitation. The shown differences in CTC size between tumor types, and between CTCs and our reference data of cultured tumor cells and patient‐derived leukocytes is striking. In conclusion, we therefore suggest that the size of CTCs does matter and should be kept in mind when designing and optimizing size‐based isolation methods.

## Conflict of interest

The authors declare no conflict of interest.

## Author contributions

PAJM, JWMM, LWMMT, and SS designed the project. PAJM, JK, and MV acquired the data. LLZ and LWMMT advised in the use of accept. EOH contributed to the statistical design. PAJM and EOH analyzed and interpreted the data. PAJM, JK, JWMM, and SS wrote, reviewed and revised the manuscript.

## Supporting information


**Fig. S1.** Number of CellSearch cartridges per tumor type.
**Fig. S2.** Number of patients per tumor type.
**Fig. S3.** ACCEPT efficiency vs. CellSearch and ACCEPT results in breast and prostate cancer.
**Fig. S4.** ACCEPT efficiency vs. CellSearch and ACCEPT results in colorectal and bladder cancer.
**Fig. S5.** Cell diameter (μm) and size (μm^2^) per cell line.
**Fig. S6.** Nucleus diameter (μm) and size (μm^2^) per cell line.

**Table S1.** Trial details of included cartridges.
**Table S2.** ACCEPT results reference data.
**Table S3.** Description of ACCEPT events in relation to CellSearch data.
**Table S4.** Nucleus singularity determination (in CK+, single CTC, DAPI+, DAPI<CK ACCEPT events).
