# Supplementary material for: Defining the dimensions of circulating tumor cells in a large series of breast, prostate, colon, and bladder cancer patients
Source: Mol Oncol. 2020 Oct 4;15(1):116–25. doi: 10.1002/1878-0261.12802 (PMC7782084; doi:10.1002/1878-0261.12802)
Supplement: Supplementary file 1 — Fig. S1. Number of CellSearch cartridges per tumor type. Fig. S2. Number of patients per tumor type. Fig. S3. ACCEPT efficiency vs. CellSearch and ACCEPT results in breast and prostate cancer. Fig. S4. ACCEPT efficiency vs. CellSearch and ACCEPT results in colorectal and bladder cancer. Fig. S5. Cell diameter (μm) and size (μm2) per cell line. Fig. S6. Nucleus diameter (μm) and size (μm2) per cell line. [file MOL2-15-116-s001.pdf]

Supplemental Figures

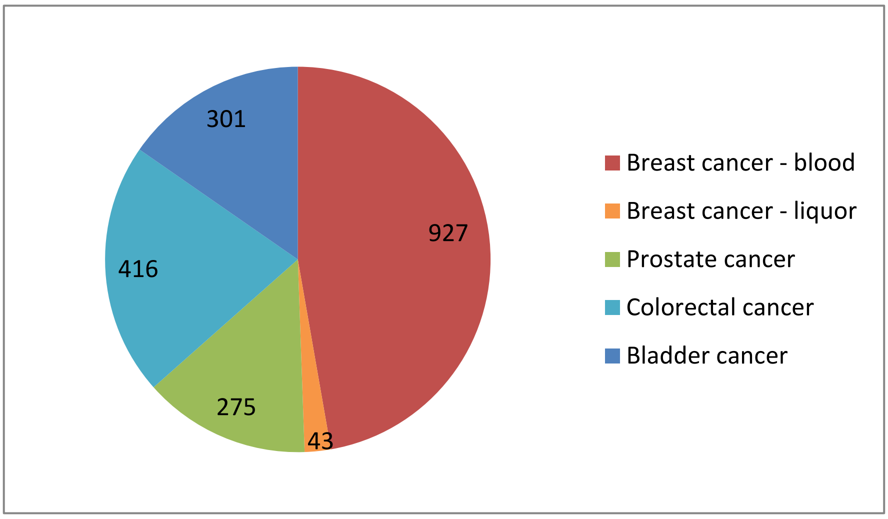

Supplemental Figure 1 - Number of CellSearch® cartridges per tumor type

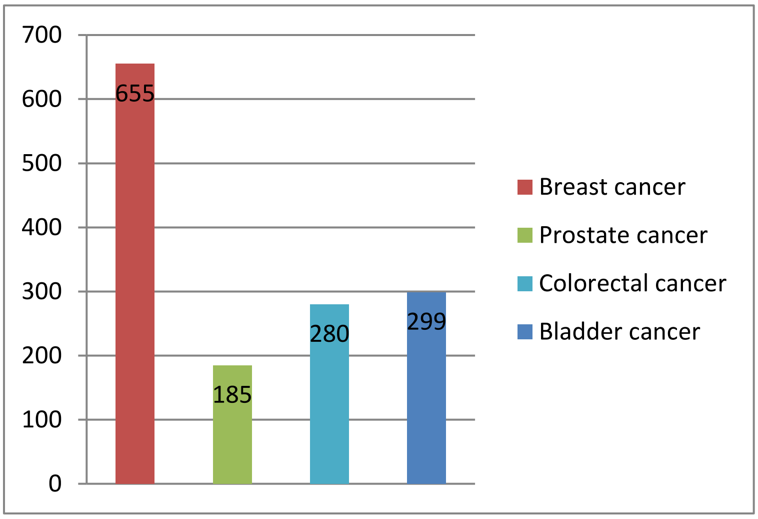

Supplemental Figure 2 - Number of patients per tumor type

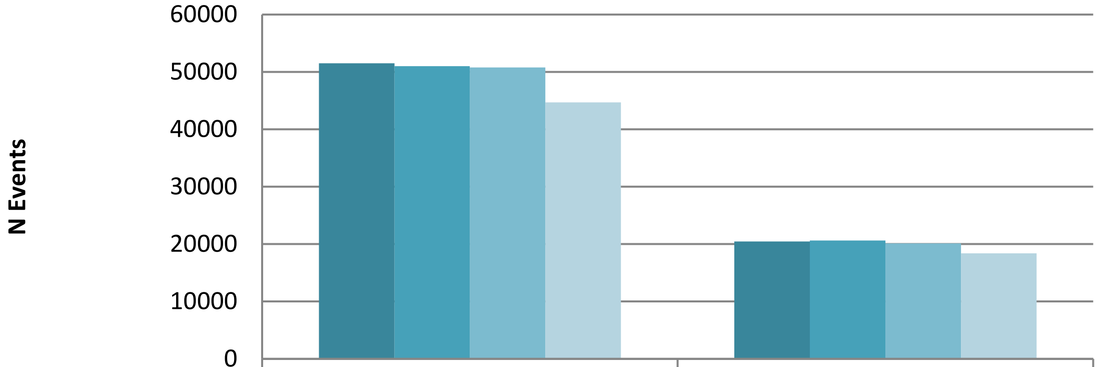

	Breast	Prostate
N CellSearch CTCs	51527	20442
N ACCEPT Events	51022	20624
N CK+ Events	50794	20100
N CK&DAPI+ Events	44672	18421

Supplemental Figure 3 – ACCEPT efficiency vs. CellSearch and ACCEPT results in breast and prostate cancer
(N= number of events detected by ACCEPT or CellSearch)

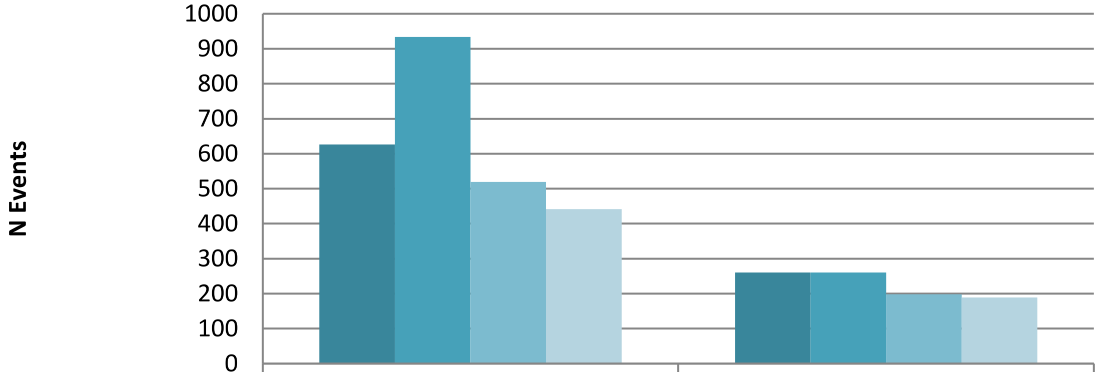

	Colorectal	Bladder
N CellSearch CTCs	626	260
N ACCEPT Events	934	260
N CK+ Events	519	198
N CK&DAPI+ Events	442	189

Supplemental Figure 4 – ACCEPT efficiency vs. CellSearch and ACCEPT results in colorectal and bladder cancer
(N= number of events detected by ACCEPT or CellSearch)

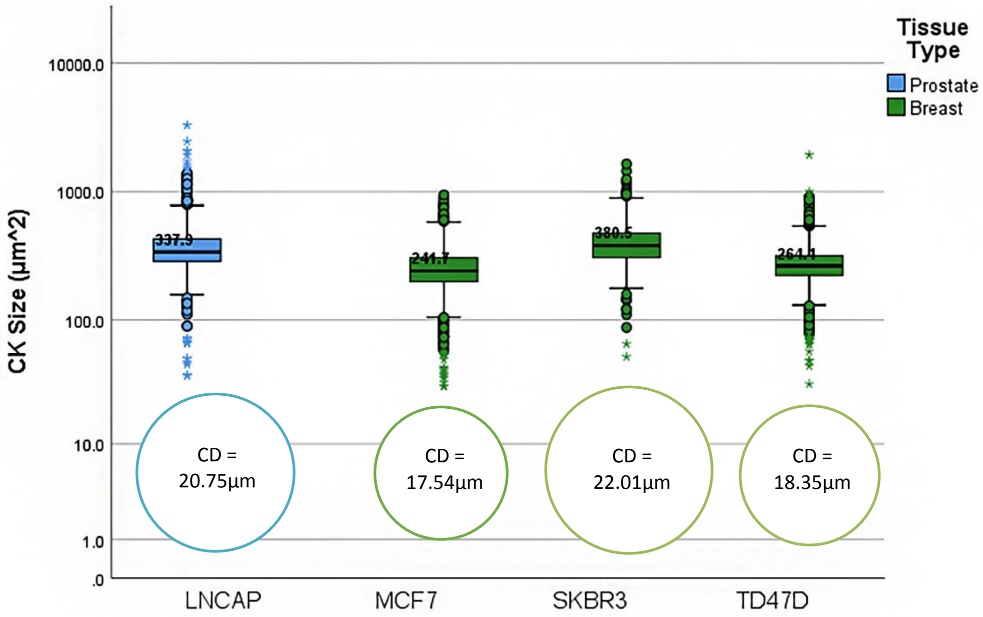

Supplemental Figure 5 – Cell diameter (µm) and size (µm²) per cell line
(Included: CK+, single CK ACCEPT events. CD: computed diameter)

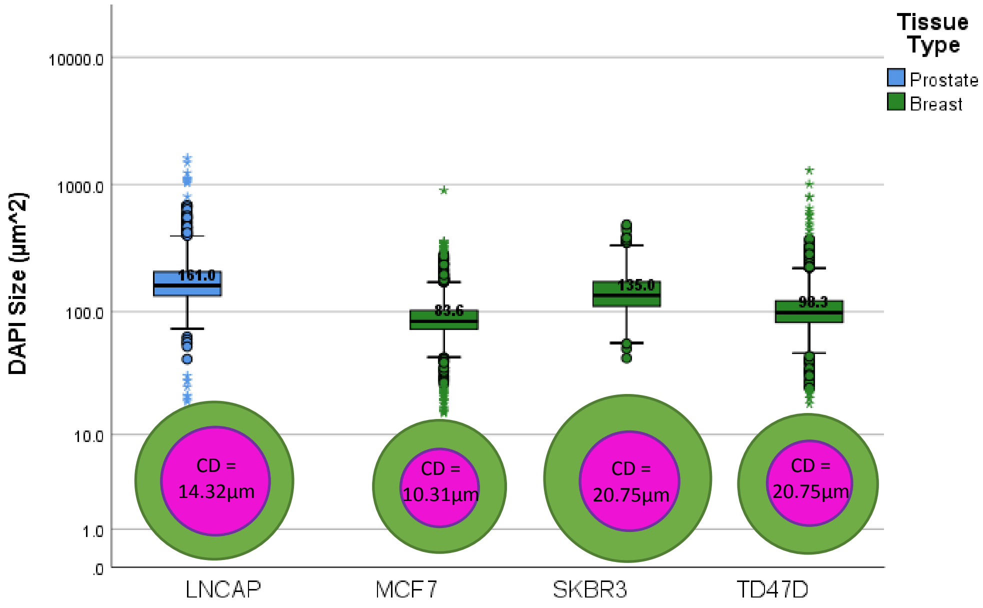

Supplemental Figure 6 – Nucleus diameter (µm) and size (µm²) per cell line
(Included: CK+, DAPI+ single CK ACCEPT events. CD: computed diameter)
