# Supplementary material for: Defining the dimensions of circulating tumor cells in a large series of breast, prostate, colon, and bladder cancer patients
Source: Mol Oncol. 2020 Oct 4;15(1):116–25. doi: 10.1002/1878-0261.12802 (PMC7782084; doi:10.1002/1878-0261.12802)
Supplement: Supplementary file 2 — Table S1. Trial details of included cartridges. Table S2. ACCEPT results reference data. Table S3. Description of ACCEPT events in relation to CellSearch data. Table S4. Nucleus singularity determination (in CK+, single CTC, DAPI+, DAPI<CK ACCEPT events). [file MOL2-15-116-s002.pdf]

Supplemental Tables

Clinical trials				
Tumor Type	Local study name	Dutch trial register number	EMC-METC	Number of included cartridges
Breast	248		MEC06-248	253
	405		MEC09-405	297
	CareMore – AI	NL4884	MEC14-588	92
	CareMore – Trastuzumab	NL4977	MEC14-589	39
	CTC-cDDP	NL3885	MEC13-007	55
	IMPACT		MEC16-313	159
	Liquor	NL5408	MEC15-419	75
Prostate	CABARESC	NL2849	MEC11-324	210
	Prelude		N.A.	41
	Process		MEC16-464	24
Colorectal	Miracle		MEC15-289	151
	RMD		MEC06-089	265
Bladder	CirGuidance	NL3954	MEC13-301	301

Supplemental Table 1. Trial details of included cartridges. For more information visit: https://www.trialregister.nl/

Origin cells	Cell type	Number of ACCEPT events
Breast cancer cell line	MCF-7	1616
	SKBR-3	530
	TD47D	1249
Prostate cancer cell line	LNCAP	1054
Patient derived leukocytes	Lymphocytes	130

Supplemental Table 2. ACCEPT results Reference data

		ACCEPT - Results		
		N Events (% of CellSearch® result)	N CK+ events (% of total ACCEPT result)	N CK+/DAPI+ events (% of total ACCEPT result)
Breast	Total	51022 (99%)	50795	44672
	Blood	45695 (94.3%)	45472 (99.5%)	39394 (86.2%)
	Liquor	5327 (162%)	5323 (99.9%)	5278 (99.1%)
Prostate		20624 (100.1%)	20100 (97.5%)	18421 (89.3%)
Colorectal		934 (149%)	519 (55.6%)	442 (47.3%)
Bladder		260 (100%)	198 (76.2%)	189 (72.69%)

Supplemental Table 3. Description of ACCEPT events in relation to CellSearch® data

	DAPI - Type of ACCEPT event					
	**Material**	**Single CTC**	**Doublet**	**Small cluster**	**Large cluster**	**Total**
**Breast**	Blood	33696	624	23	0	34343
	Liquor	4515	58	2	0	4575
**Prostate**	Blood	13127	224	5	0	13356
**Colorectal**	Blood	252	3	0	0	255
**Bladder**	Blood	93	4	2	0	99
**Total**		51683	913	32	0	52628

Supplemental Table 4. Nucleus singularity determination (in CK+, single CTC, DAPI+, DAPI<CK ACCEPT events)
